# Sleep, Well-Being, and Cognition in Medical Interns on a Float or Overnight Call Schedule

**DOI:** 10.1001/jamanetworkopen.2024.38350

**Published:** 2024-10-11

**Authors:** Stijn A. A. Massar, Xin Yu Chua, Ruth Leong, Hosein A. Golkashani, Zhenghao Pu, Alyssa S. C. Ng, Ju Lynn Ong, Chun Siong Soon, Nicholas B. H. Ng, Mae Yue Tan, Jeremy B. Lin, Marion Aw, Michael W. L. Chee

**Affiliations:** 1 Sleep and Cognition Laboratory, Centre for Sleep and Cognition, Yong Loo Lin School of Medicine, National University of Singapore, Singapore, Singapore; 2 Department of Paediatrics, Khoo Teck Puat—National University Children’s Medical Institute, National University Health System, Singapore, Singapore; 3 Department of Paediatrics, Yong Loo Lin School of Medicine, National University of Singapore, Singapore, Singapore

## Abstract

**Importance:**

Extended work hours and night shifts are essential in health care, but negatively affect physician sleep, well-being, and patient care. Alternative schedules with shorter work hours and/or reduced irregularity might mitigate these issues.

**Objective:**

To compare sleep, well-being, and cognition between interns working irregular, extended shifts (call schedule), and those working a more regular schedule with restricted hours (float schedule).

**Design, Setting, and Participants:**

In this observational longitudinal cohort study, interns in a Singapore-based teaching hospital were studied for 8 weeks from January 2022 to July 2023. Data were analyzed from July 2023 to July 2024.

**Exposure:**

Participants worked either regular approximately 10-hour workdays, interspersed with 24 hour or more overnight calls 4 to 5 times a month, or a float schedule, which included regular approximately 10-hour workdays, and 5 to 7 consecutive approximately 12-hour night shifts every 2 months. Exposure was based on departmental training and operational needs.

**Main Outcomes and Measures:**

Sleep was measured with wearable sleep trackers and an electronic diary. Day-to-day well-being and cognitive assessments were collected through a smartphone application. Assessments included the Sleep Regularity Index (SRI; determines the probability of an individual being in the same state [sleep or wake] at any 2 time points 24 hours apart, with 0 indicating highly random sleep patterns and 100 denoting perfect regularity) and Pittsburgh Sleep Quality Inventory (PSQI; scores ranges from 0 to 21, with higher scores indicating poorer sleep; a score greater than 5 suggests significant sleep difficulties).

**Results:**

Participants (mean [SD] age, 24.7 [1.1] years; 57 female participants [59.4%]; 41 on call schedule [42.7%]; 55 on float schedule [57.3%]) provided 4808 nights of sleep (84.2%) and 3390 days (59.3%) of well-being and cognition assessments. Participants on a float schedule had higher SRI scores (mean [SD] score, 69.4 [6.16]) and had better quality sleep (PSQI mean [SD] score, 5.4 [2.3]), than participants on call schedules (SRI mean [SD] score, 56.1 [11.3]; *t*_91_ = 6.81; mean difference, 13.3; 95% CI, 9.40 to 17.22; *P* < .001; PSQI mean [SD] score, 6.5 [2.3]; *t*_79_ = 2.16; 95% CI, 0.09 to 2.15; *P* = .03). Overnight call shifts, but not night float shifts, were associated with poorer mood (−13%; β = −6.79; 95% CI, −9.32 to −4.27; *P* < .001), motivation (−21%; β = −10.09; 95% CI, −12.55 to −7.63; *P* < .001), and sleepiness ratings (29%; β = 15.96; 95% CI, 13.01 to 18.90; *P* < .001) and impaired vigilance (21 ms slower; β = 20.68; 95% CI, 15.89 to 25.47; *P* < .001) compared with regular day shifts. Night shifts with naps were associated with better vigilance (16 ms faster; β = −15.72; 95% CI, −28.27 to −3.17; *P* = .01) than nights without naps.

**Conclusions and relevance:**

In this cohort study, 24-hour call schedules were associated with poorer sleep, well-being, and cognition outcomes than float schedules. Naps during night shifts benefited vigilance in both schedules.

## Key Points

**Question:**

Is a night float schedule with consecutive 12-hour night shifts associated with reduced burden on intern sleep, well-being, and performance compared with a 24-hour overnight call schedule?

**Findings:**

In this cohort study of 96 participants, tracking across 8 weeks showed more regular and better-quality sleep on a float schedule than on a call schedule. A 24-hour overnight call was adversely associated with mood, motivation, sleepiness, and impaired cognition; participants on the float schedule did not have these associations, and naps benefitted vigilance in both schedules.

**Meaning:**

These findings suggest a float schedule may have fewer negative outcomes for sleep, well-being, and cognition than a call schedule, and that naps during night work may be beneficial.

## Teaser

This cohort study evaluates sleep, well-being, and cognition between interns working irregular, extended shifts and those working a more regular schedule with restricted hours.

## Introduction

Extended work hours and night shifts disrupt sleep and circadian rhythms^1,2^ but are essential for the continuity of high-quality health care. The first year of postgraduate medical training engages physicians in long duty hours and overnight shifts that negatively impact sleep and neurocognitive function, as well as physical and mental health.^3,4,5,6,7,8,9,10^ To reduce physician workload while preserving educational and patient care goals, alternative schedules have been designed.^11,12,13,14,15,16^

On a traditional call schedule, interns work 24-hour or longer shifts (overnight call) in addition to regular daytime work. A popular alternative is the float schedule, where multiple consecutive night shifts are strung together (night float week), with no daytime work (maximum 16 hours continuous shift). This allows physicians to rest between night shifts and to adapt their sleep schedules over consecutive nights.

Following duty hour reforms, many US hospitals have adopted restricted duty hour shifts (16-hour shifts) for interns.^15^ However, equivocal data on their effectiveness have led the Accreditation Council for Graduate Medical Education to reverse an earlier mandate and allow for continuous shifts of a maximum of 24 hours.^16,17,18,19^ Moreover, 24-hour shifts are common in many countries worldwide.^20^

Detailed studies are needed to inform the strategic implementation of such schedules. However, continuous objective monitoring of sleep and performance is difficult to achieve, given the demanding schedules and high workload.^21^ Advances in wearable health tracking and smartphone-based ecological momentary assessments (EMA) have made the long-term intensive monitoring of sleep, cognitive performance, and mental well-being more feasible.^22,23,24,25,26^ Here, we monitored first year postgraduate (PGY1) physicians through 8 weeks of either a traditional call schedule or a float schedule using a wearable sleep tracker and smartphone-based EMAs.

The objectives of this study were to compare sleep (duration, regularity, quality, and sleepiness), well-being (mood, motivation, and stress), and cognitive performance (speed of processing, working memory, and vigilance) between interns who worked on a call schedule vs those who worked on a float schedule. We hypothesized that more beneficial outcomes would be observed for the float group compared with the call group in all these domains. Importantly, the daily sampling of these outcomes over 8 weeks per participant allowed us to compare night shifts (overnight call or night float shifts) with each person’s baseline on regular day shifts.

## Methods

### Recruitment

Incoming interns were recruited from a large training hospital in Singapore (National University Hospital) from departments that followed either a traditional call schedule (Paediatrics, Obstetrics, or Gynaecology) or a float schedule (Internal Medicine, General Surgery, or Orthopaedics). Participants were assigned to departments before recruitment in our study. Assignment was established by a centrally coordinated national agency based on indicated preference, staffing requirements, and availability of training positions. Each department followed only 1 schedule type based on department-level operational considerations, such as staffing and work requirements.

All procedures were approved by the National University of Singapore institutional review board and all participants signed informed consent before data collection. Out of a total of 326 interns, 98 consented to study participation. Two participants who initially consented withdrew before data collection. This report follows the Strengthening the Reporting of Observational Studies in Epidemiology (STROBE) reporting guidelines for cohort studies.

### Shift Schedules: Call vs Float Schedules

Participants were recruited from departments that followed either a traditional call schedule (from here onwards termed the call group) or a float schedule (float group). In the call group, interns worked from 7 am to 5 pm on most days.^21^ For 5 to 7 days a month, they performed an overnight call shift that commenced at 7 am and ended between 8 am to 1 pm the following day (with the rest of the day off). In the float group, interns worked from 7 am to 5 pm every day for most weeks. During designated periods occurring once every 2 to 3 months (night float week), they worked for 5 to 7 consecutive nights (night float shift) from 8 pm to 8 am.

### Data Collection Protocol

Upon study enrollment, participants provided demographic data (sex, age, ethnicity, height, weight, and body mass index [BMI]) through self report. Ethnicity data was collected as part of standard practice in Singapore (categories: Chinese, Malay, Indian, or Other). Over 8 weeks, participants recorded their sleep and physical activity using an Oura Ring 3 wearable device (Oura Health). Additionally, they completed daily well-being and cognitive assessments on an EMA smartphone application (Z4IP, Sleep and Cognition Laboratory) and reported their daily activities in an electronic time-use diary embedded in this application. EMA sessions were completed once between 5 am and 10 pm, to allow for completion on irregular work schedules. Data collection through wearable and phone-based methods allowed for high-resolution sampling of passive and active measures, while minimizing the participant’s burden. In cases where no night float shifts occurred within the 8 weeks (eg, due to rescheduling of the night float week), the data collection period was extended. Participants were financially remunerated for participation (up to $230 depending on completion rates).

### Sleep Measurement

**Figure 1.**
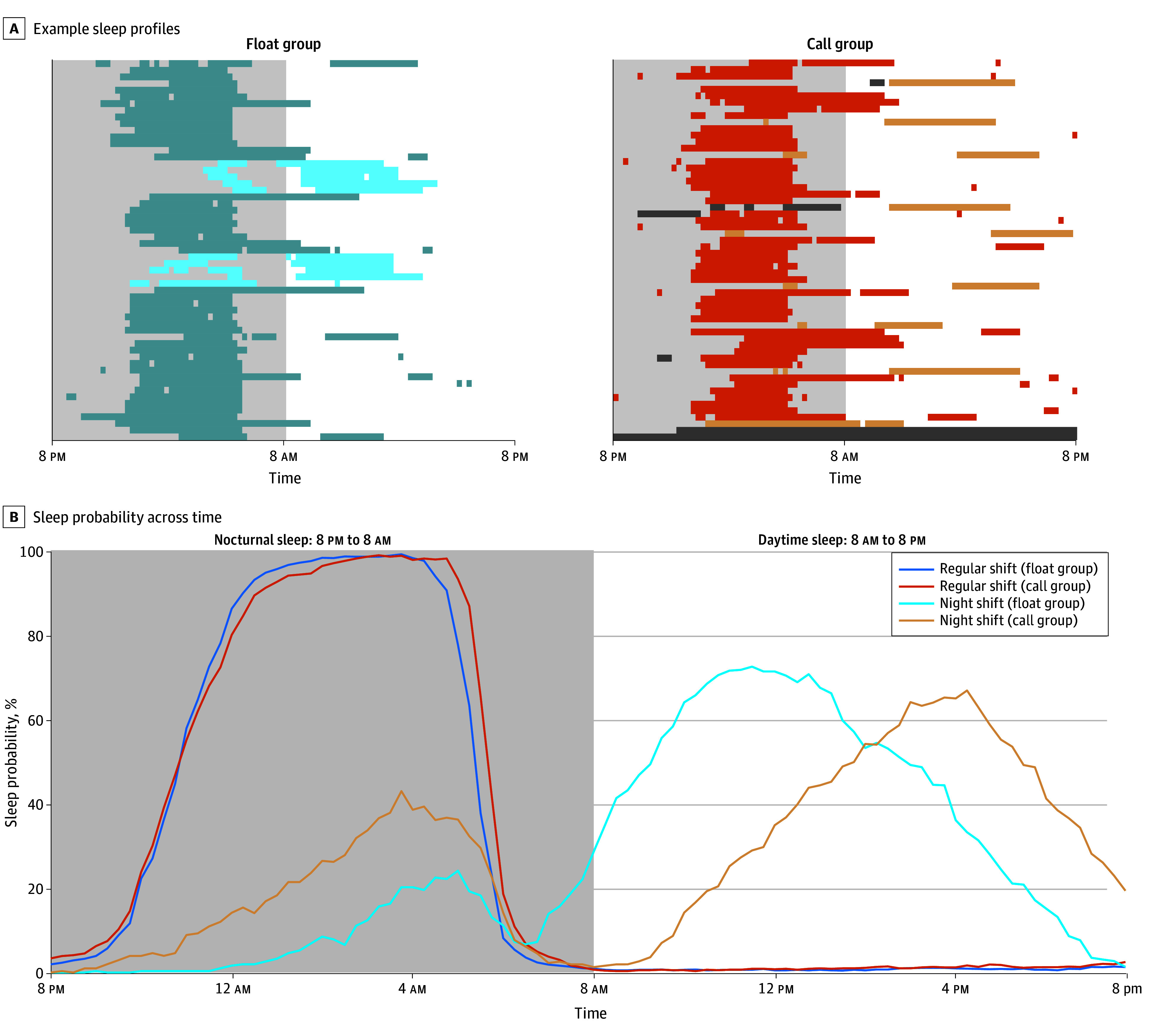
Integrated Sleep Probability Timelines A, Example sleep patterns from a float (blue) and a call participant (orange), with darker shades indicating sleep on regular shift days and lighter shades indicating sleep on night-shift days. B, Mean sleep probability over time for regular and night-shift days. Dark gray shaded areas indicate missing data. Light gray areas indicate nocturnal sleep periods, and white areas indicate daytime sleep periods.

For high-fidelity assessment of sleep, we integrated wearable-derived sleep and physical activity measurements with electronic time-use diary input (Figure 1). This enabled us to (1) impute missing data from wearable or e-diary channels, and (2) arbitrate when there was discrepancy between different channel outputs, such as detecting short naps not detected by the wearable.^27^ Details of this procedure are described in eFigure 1 in Supplement 1. Briefly, an automated pipeline was created that calculated the proportion of time spent asleep in 15-minute time windows for each channel (wearable device sleep, wearable device activity, and sleep diary). These proportions were combined into a sleep probability score (ranging from 0 = likely wake to 1 = likely sleep), where the contribution of each channel was weighted by its correlation with the other channels. For each day, sleep duration was computed separately for nocturnal (8 pm to 8 am) and daytime periods (8 am to 8 pm). Only periods with at least 75% data were included for analyses. Furthermore, the Sleep Regularity Index (SRI)^28^ was calculated, which determines the probability of an individual being in the same state (sleep or wake) at any 2 time points 24 hours apart. A score of 0 indicates highly random sleep patterns, while 100 denotes perfect regularity.

### Daily Cognitive and Well-Being Assessment

Each day, participants completed a short cognitive and well-being assessment through an EMA mobile application (details in eFigure 2 in Supplement 1). In brief, each session comprised 3 short cognitive tasks evaluating speed of processing (symbol search task), working memory (dot-memory task), and vigilance (abbreviated psychomotor vigilance test [PVT-B]).^29^ Following this, participants filled in their bedtime, wake time, and sleep quality (5-point scale with 1 indicating very poor and 5 indicating very good) for the preceding sleep period. They also indicated their current mood (sliding scale with 0 indicating negative and 100 indicating positive), sleepiness, stress, motivation, and loneliness (sliding scale with 0 indicating not at all and 100 indicating very much) levels.

### 10-Minute PVT

To validate the EMA-delivered 3-minute PVT, a subset of participants additionally completed the more established 10-minute PVT on a laptop computer.^30^ Participants completed 3 PVT sessions on days after their night shift (between 8 am and 2 pm; before recovery sleep), and 3 control sessions after a full night of sleep. The median of the individual reaction time distribution (medRT), attentional lapses (responses >500ms), and false starts (responses before target onset or <150 after target onset) were extracted as outcome metrics.

### Statistical Analysis

The analysis specifically focused on comparing sleep, well-being, and cognitive performance between the different schedule groups (call group vs float group), on the different shift events during their posting (regular day shifts vs night shifts [ie, overnight call shift for the call group or night float shift for the float group]). Linear mixed models (LMM) were constructed with the factors group (call or float), shift (regular day shift or night shift), and their interaction. Where a significant group × shift interaction was found, pairwise comparisons were calculated to estimate the difference between groups for each regular shift and night shift separately. All models controlled for participant demographics (age, gender, and BMI), and for any changes over the study period (day-of-study). To control for various possible influencing factors, a set of control analyses were performed. In control analysis 1, the variable timing of the well-being and cognitive assessments was accounted for. Control models were constructed with total sleep duration in the prior 24 hours, and time awake since the last sleep episode as additional control variables. Control analysis 2 examined whether outcomes associated with well-being and/or cognition were present before the start of a work shift, or whether they developed over the course of the shift by selecting EMA sessions completed before the work shift (preshift) or after work shift (postshift). Separate LMMs were constructed with group (call or float), shift (regular days shift or night shift), and timing (preshift or postshift), while controlling for demographics and day-of-study. Control analysis 3 specifically assessed whether the outcomes associated with night shifts could be mitigated when interns had an opportunity to sleep during their night shift. Postshift assessments for night shifts with and without an on-shift nap were compared. Lastly, performance on the 10-minute PVT was examined with an LMM using group (call, float) and day (control, night shift) as factors, and controlling for demographics and day-of-study. All analyses were conducted in SPSS version 29.0.1.0(171) (IBM Corp). Statistical tests were corrected for multiple comparisons using the Benjamini-Hochberg method,^31^ with corrected significance threshold set to a 2-sided α = .05. Data were analyzed from July 2023 to July 2024.

## Results

### Sample Characteristics

Demographics and baseline characteristics for the 96 participants (mean [SD] age, 24.7 [1.1] years; 57 female participants [59.4%]) are displayed in Table 1. Forty-one participants were on a call schedule and 55 participants were on a float schedule. The call and float groups did not significantly differ in age, ethnicity, and BMI. However, there were more female participants in the call group (31 participants [75.6%]) than in the float group (26 participants [47.3%]). The 2 groups had similar sleep quality, chronotype, and self-rated depression and burnout measures at study commencement. Over the study period, participants on a call schedule reported a mean (SD) of 64.4 (8.52) work hours per week, with a mean (SD) of 8.6 (2.0) night shifts (overnight call shifts). Participants on a float schedule, over their observed period, reported a comparable number of working hours per week (mean [SD], 64.2 [8.06] hours), with about 1 night float week during the study period (mean [SD], 6.2 [2.3] night shifts per night float week). For 10 participants from the float group, no night float week had occurred within the original study period, and data collection was extended (mean [SD], 2.7 [1.9] weeks extension).

**Table 1.** Sample Demographics and Baseline Characteristics for the Night Call and Float Groups

Characteristic	Participants, No (%)			*t *Value, *U*-statistic, or χ^2^
	Overall	Call group	Float group	
Age, median (IQR), y	24.3 (24.0-25.1)	24.3 (23.9-25.0)	24.3 (24.0-25.4)	*U* = 1079
Sex				χ^2^ = 7.82^a^
Female	57 (59.4)	31 (75.6)	26 (47.3)	
Male	39 (40.6)	10 (24.4)	29 (52.7)	
Ethnicity				χ^2^ = 2.86
Chinese	88 (91.7)	38 (92.7)	50 (90.9)	
Non-Chinese	8 (8.3)	3 (7.3)	5 (9.1)	
Body mass index, median (IQR)^b^	20.8 (19.2-22.8)	20.8 (19.2-21.9)	21.0 (19.2-23.1)	*U* = 1032
Baseline characteristics				
Sleep quality (PSQI), mean (SD)	5.8 (2.3)	5.9 (2.4)	5.8 (2.3)	*t* = 0.24
Chronotype (MEQ), mean (SD)	49.4 (7.6)	48.6 (6.6)	49.9 (8.2)	*t* = 0.76
Depression (BDI), median (IQR)	10.0 (5.0-16.8)	10.0 (5.0-14.0)	11.0 (5.3-18.0)	*U* = 843
Burnout (OLBI), mean (SD)	43.1 (6.8)	42.3 (6.1)	43.6 (7.2)	*t* = 0.92

Abbreviations: BDI, Beck Depression Inventory; MEQ, Morningness-Eveningness Questionnaire; OLBI, Oldenburg Burnout Inventory; PSQI, Pittsburgh Sleep Quality Inventory.

^a^ *P* < .01.

^b^ Calculated as weight in kilograms divided by height in meters squared.

### Sleep Outcomes

A total of 4808 days (84.2%) of sleep data met analysis criteria (mean [SD], 50.05 [12.54] days/person). Figure 1A shows example sleep profiles from 2 participants (float vs call schedule). Figure 1B shows the mean sleep probability for regular day shifts (starting from the night preceding the workday) and for night shifts. On regular days, a consolidated bout of nocturnal sleep was observed in both groups, while on night shift days, distinct bouts of nocturnal sleep (during the night shift hours) and recovery sleep (in the daytime after the night shift) were observed. LMMs were used to compare sleep duration between groups (call or float) and shifts (regular days or night shift).

#### Sleep Duration and Regularity

**Figure 2.**
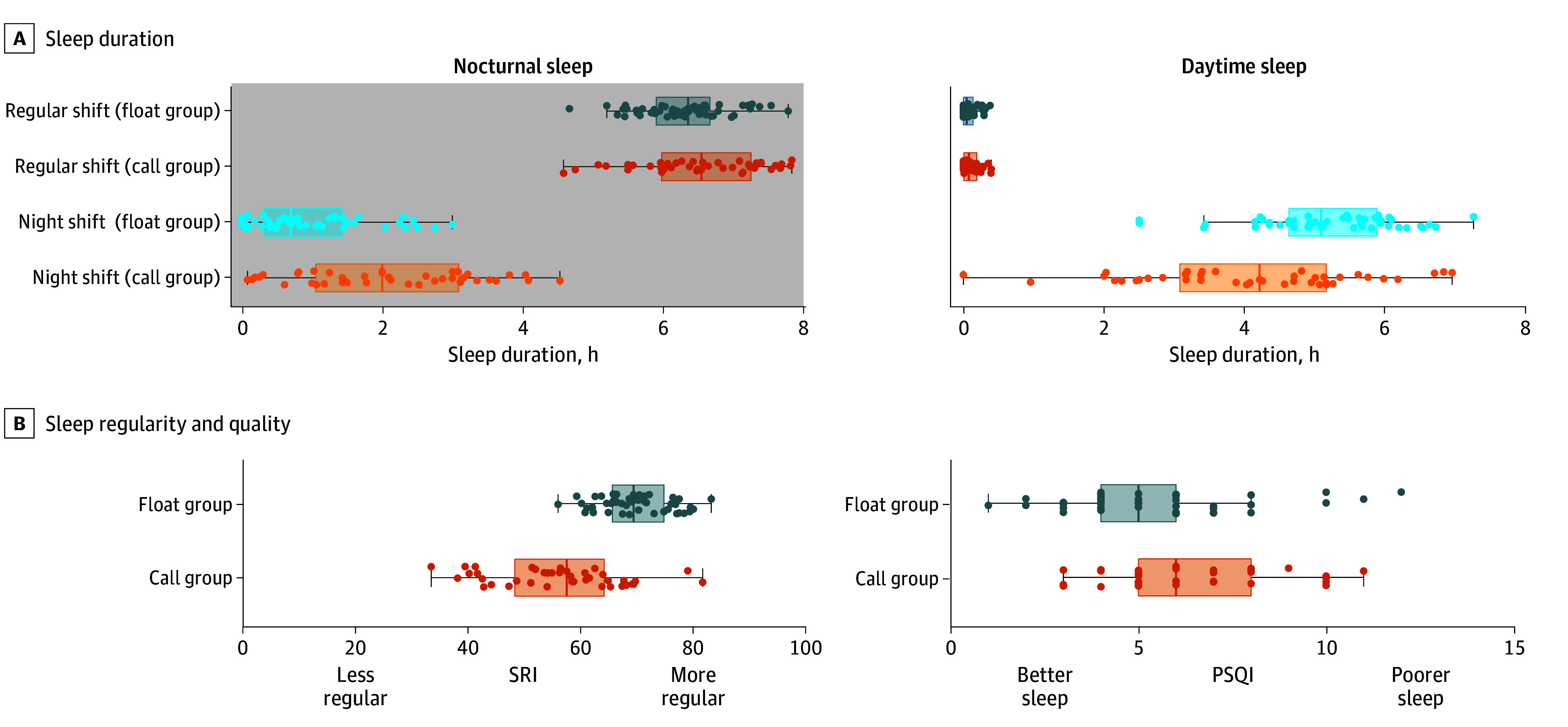
Group-Level Sleep Outcomes for Call Group And Float Group A, Sleep duration over the nocturnal (left: 8 pm – 8 am) and daytime periods (right: 8 am to 8 pm). B, SRI and PSQI scores. Boxes represent median and IQR. Error bars represent 2 minimum and maximum excluding outliers. Dots represent individual mean scores. PSQI indicates Pittsburgh Sleep Quality Inventory; SRI, Sleep Regularity Index.

Nocturnal sleep duration showed a group × shift interaction (β = 48.11; 95% CI, 35.67-60.54; *P* < .001) (Figure 2A). On regular days, participants in the call group had a mean of 16.98 minutes longer nocturnal sleep (mean [SE], 6.6 hours [6.32 minutes]) than the float group (mean [SE], 6.34 hours [5.04 minutes]; β = 16.98; 95% CI, 0.83 to 33.13; *P* = .04), with no differences for daytime sleep (call group mean [SE], 7.13 [3.30] minutes; float group mean [SE], 5.02 [2.57] minutes; β = 2.11; 95% CI, −6.22 to 10.45; *P* = .62). On night shifts, nocturnal sleep indicated naps taken during the work shift. The call group obtained longer naps during their night shift (mean [SE], 2.08 hours [7.06 minutes]) than the float group (mean [SE], 59.78 [6.17] minutes; β = 65.09; 95% CI, 46.51 to 83. 67; *P* < .001). In contrast, during the daytime following night shifts, the float group obtained more sleep (mean [SE], 5.16 hours [3.92 minutes]) than the call group (mean [SE], 4.22 hours [4.27 minutes]; β = 56.20; 95% CI, 44.79 to 67.62; *P* < .001). Across all qualifying 24-hour periods analyzed, interns in the float group had significantly higher sleep regularity (SRI mean [SD], 69.4 [6.16]) (Figure 2B) than the call group (mean [SD], 56.1 [11.3]; *t*_91_ = 6.81; *P* < .001).

#### Questionnaires

Participants in the call group reported poorer subjective sleep quality (PSQI mean [SD], 6.5 [2.3]) compared with the float group (mean [SD], 5.4 [2.3]; *t* = 2.16; *P* = .03) (Figure 2B). However, no significant differences in depression, burnout, or shift work–related insomnia were reported 8 weeks after living under these schedules (Table 2).

**Table 2.** Well-Being Questionnaires at Study Exit (After 8 Weeks of Night Call and Night Float Schedule Work)

Questionnaire	Mean (SD)		*t *Value	*P* value
	Call group	Float group		
Sleep quality (PSQI)	6.5 (2.3)	5.4 (2.3)	2.16	.03
Depression (BDI)	10.5 (8.1)	10.6 (10.1)	0.06	.96
Burnout (OLBI)	41.0 (6.5)	42.4 (7.1)	0.87	.20
Bergen Shift Work Sleep Questionnaire				
Day shifts	10.70 (3.37)	9.63 (3.21)	−1.45	.15
Night shifts	12.10 (3.91)	11.13 (4.28)	−0.99	.32
Rest days	7.00 (3.43)	5.67 (2.72)	−1.91	.06

Abbreviations: BDI, Beck Depression Inventory; OLBI, Oldenburg Burnout Inventory; PSQI, Pittsburgh Sleep Quality Inventory.

### Daily Well-Being and Cognitive Assessments

**Figure 3.**
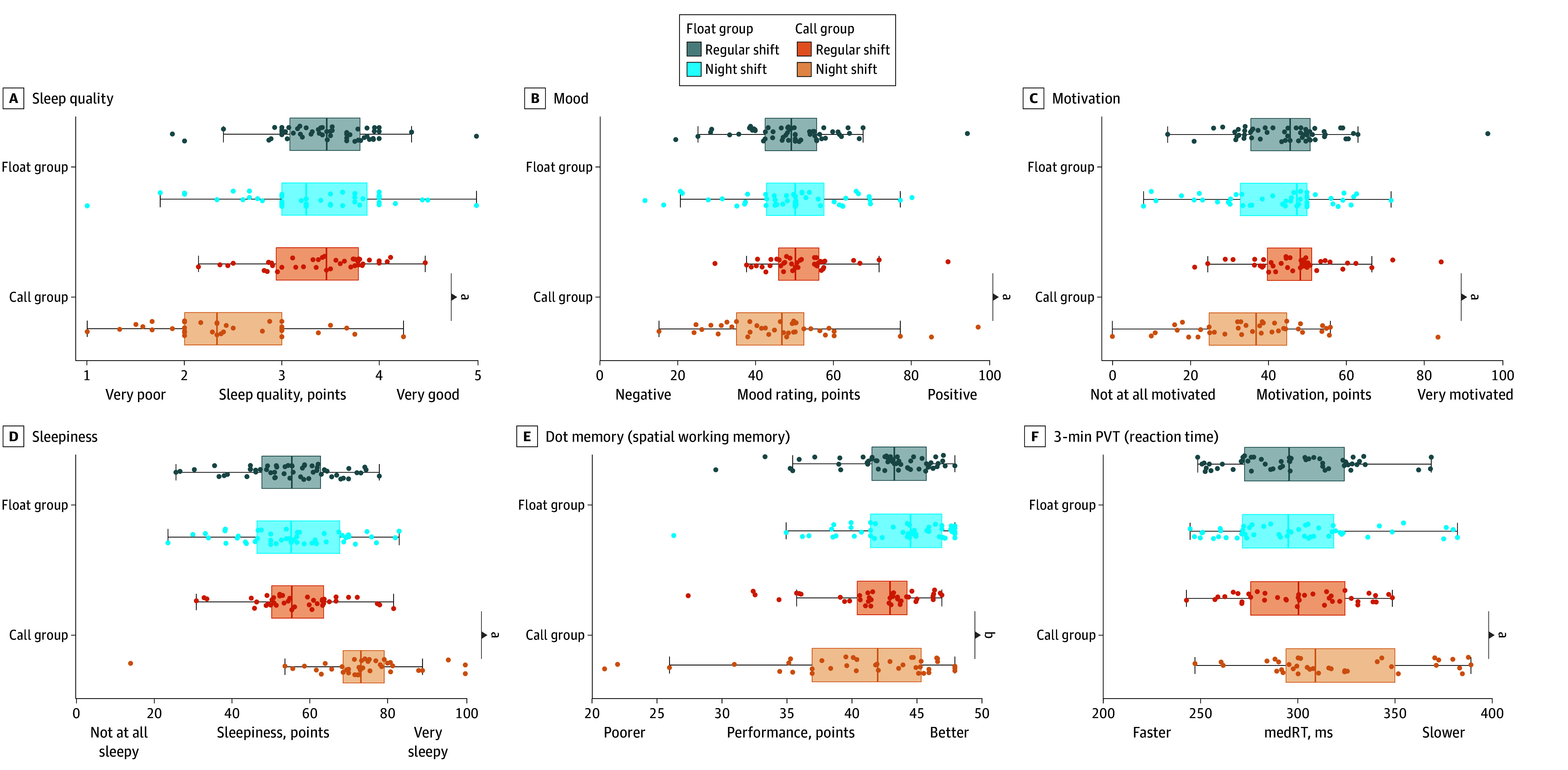
Mean Well-Being Scores and Cognitive Performance Recorded by Daily Phone-Based Assessment Boxes represent median and IQR. Error bars represent 2 minimum and maximum excluding outliers. Dots represent individual mean scores. PVT indicates abbreviated psychomotor vigilance test. ^a^*P* < .001. ^b^*P* < .05.

Linear mixed models yielded significant group × shift interactions for self-reported sleep quality (Figure 3) (β = −0.89; 95% CI, −1.05 to −0.73), sleepiness (β = 14.82; 95% CI, 10.96 to 18.69), mood (β = −6.56; 95% CI, −9.88 to −3.25), and motivation (β = −8.31; 95% CI, −11.54 to −5.08). Pairwise-comparisons showed that following an overnight call shift, participants reported poorer sleep quality (β = −0.98; 95% CI, −1.11 to −0.86; *P* < .001) (eTable 1 and eTable 2 in Supplement 1), increased sleepiness (β = 15.96; 95% CI, 13.01 to 18.90; *P* < .001), poorer mood (β = −6.79; 95% CI, −9.32 to −4.27; *P* < .001) (eTable 3 and eTable 4 in Supplement 1), and lower motivation (β = −10.09; 95% CI, −12.55 to −7.63; *P* < .001) relative to their regular day shifts. For float participants, these ratings were not significantly different between regular day shifts and night float shifts (sleep quality: β = −0.094; 95% CI, −0.19 to 0.003; *P* = .06; sleepiness: β = 1.1; 95% CI, −1.38 to 3.65; *P* = .38; mood: β = −0.23; 95% CI, −2.39 to 1.93; *P* = .83; motivation: β = −1.8; 95% CI, −3.88 to −0.32; *P* = .10). No significant interactions were found for stress and loneliness ratings (eTable 5 and eTable 6 in Supplement 1).

For cognitive performance, significant group × shift interactions were found for the working memory (β = −1.42; 95% CI, −2.52 to −0.31) and vigilance tasks (β = 21.01; 95% CI, 14.80 to 27.21). For both tasks, performance was worse after the overnight call shift compared with regular days for the call group (working memory: β = −1.11; 95% CI, −1.95 to −0.26; *P* = .01; PVT: β = 20.68; 95% CI, 15.89 to 25.47; *P* < .001) but not for the float group (night float shift vs regular days nonsignificant). No significant main effects of group or shift were found for the speed of processing tasks (eTable 7 and eTable8 in Supplement 1).

### Control Analyses

As EMA reports could be made from 5 am to 10 pm, to allow for completion on irregular work schedules, 2 sets of control analyses were performed (eFigure 3 in Supplement 1). First, we ran a set of linear mixed models with total sleep duration in the prior 24 hours and time awake since the last sleep episode as control variables. Importantly, while prior sleep duration explained some variance in sleep ratings (sleep quality and sleepiness), well-being (mood and motivation), and cognition (working memory and vigilance), the negative outcomes of night shifts on the call group (but not float group) remained significant (eTable 1, eTable 2, eTable 3, eTable 4, eTable 5, eTable 6, eTable 7, and eTable 8 in Supplement 1).

Second, we performed LMM analyses comparing the EMA sessions that were completed before starting the work shift, with sessions completed at the end of the work shift. These analyses showed that for most measures, detrimental outcomes were particularly observed at the end of night shifts (eTable 9, eTable 10, eTable 11, eFigure 4, and eFigure 5 in Supplement 1). Following up on this, the outcome of naps taken during night shifts on vigilance was examined by comparing night shifts that included a nap vs those that did not include a nap. Results showed that vigilance performance was better after a night shift with a nap (mean [SE] medRT, 309.1 [4.94] ms) than after a night shift without a nap (mean [SE] medRT = 324.8 [6.76] ms) in both groups (nap main effect: β = −15.72; 95% CI, −28.27 to −3.17; *P* = .01 with no nap × group interaction: β = 12.16; 95% CI, −12.91 to 37.22; *P* = .34) (eTable 12 and eFigure 6 in Supplement 1).

### 10-Minute Psychomotor Vigilance Task

Seventy-three participants (34 in the call group and 39 in the float group) additionally performed a standardized 10-minute PVT^30^ (3 times after a full night of sleep [control days] and 3 times after a night shift). Both groups had poorer performance after their night duties compared with control days. However, this impairment was larger in the call group than in the float group (group × shift interaction: lapses, β = −5.56; 95% CI, −8.60 to −2.51; median RT: β = −40.92; 95% CI, −77.30 to −4.53; false starts: β = −1.29; 95% CI, −2.24 to −0.33) (eTable 13 and eFigure 7 in Supplement 1).

## Discussion

Over 8 weeks, the float schedule was associated with more regular sleep patterns and better self-reported sleep quality compared with the call schedule. Additionally, day-to-day mental well-being and cognitive assessments were relatively unperturbed after night float shifts, while 24-hour overnight call shifts were associated with poorer sleep and well-being ratings and impaired cognitive performance (poorer working memory and vigilance).

The call and float schedules differed in several key aspects. First, the call schedule required interns to work extended hours (≥24 hours vs 12 hours on night float) with associated accumulation of sleep deprivation and fatigue. Second, it involved frequent rotations from day to night shifts (ie, 8 to 10 shifts over the 8-week study period). In the float schedule, night shifts were concentrated in a week occurring once or at most twice over the same period, while the majority of weeks consisted of regular day shifts.

Although the call group in our study had equal or longer sleep duration compared with the float group, their sleep schedule was characterized by higher sleep irregularity. Sleep irregularity is increasingly recognized as a risk factor for health and well-being.^32,33^ In the short term, sleep irregularity has been associated with mental health issues in the general population,^34,35,36^ as well as in physicians.^22^ Longer term, high sleep irregularity raises the risk for cardiovascular disease, hypertension, and premature death^37,38^ more than short sleep duration.^39^

Accordingly, daily ratings of mood, motivation, and sleepiness were worse after an overnight call shift. This triad has been associated with an individual’s readiness to perform in relation to the prior night’s sleep duration^40,41^ and could affect learning and interpersonal interactions. Relatedly, physician empathy has a positive influence on patient perceptions about the professionalism of their care,^42^ and it is diminished by fatigue. Reduction of empathy in association with night work could also alter therapeutic decision-making; for example, being less willing to prescribe analgesics to alleviate pain.^43^

Objective assessment of working memory and vigilance were also relatively poorer after overnight calls, confirming prior findings.^4,8,10^ Importantly, impaired working memory has been associated with medical errors in the face of sleep loss.^44^ Impaired vigilance relates to missing critical signals^45^ and involuntarily falling asleep.^46^ Given that the greatest impairments were found at the end of night shifts, this may not only relate to on-duty functioning, but may also confer a risk immediately postduty, affecting interns who may have to drive home.^47,48^ As supported by our data, on-shift napping may mitigate the vigilance deficits associated with night shifts.^49,50^

Patient outcomes and educational outcomes were not collected in this study. It has been suggested that long duty hours may allow for more professional education and decrease adverse patient outcomes arising from more frequent hand offs.^51^ Against these concerns, a recent study involving PGY1 interns in 2 training hospitals in Singapore examined official records of educational outcomes and medical errors. No significant differences in learning attainment or patient safety were found.^52^ A separate nationwide survey study reported that 78.9% of interns found a float schedule less disruptive to learning practice and 86.9% felt that a float schedule helped to reduce medical errors.^53^

### Strengths and Limitations

The current study has several strengths. Objectively assessing sleep by combining wearable and smartphone-based sources of sleep measurement reduced the impact of missing data. Also, as wearable sleep measurement algorithms have not been designed for persons with irregular sleep schedules, this approach provided superior sleep assessments by complementing the strengths of different sources of sleep information.^27,54,55,56,57^

The regular sampling of mental well-being, and cognitive performance outcomes throughout the interns’ different shift events is unprecedented in participants on busy schedules, such as medical interns. Evaluation over 8 weeks provided a representative depiction of interns’ day-to-day experiences across a mixture of regular and night-shift nights. While only first-year interns were included in this study, it could be expected that the effects of the float and call schedules on sleep, well-being and cognitive performance extend to other junior physicians working similar schedules.

The most notable limitation of this study is the observational nature of the design. Exposure conditions were not randomly assigned but were determined by the departments to which participants were allocated. The findings should be considered associations. Additional limitations include the inherently different work requirements between departments (for example, pediatrics vs surgery), although both exposure groups were composed of multiple specialties. Moreover, the day-to-day sampling allowed us to examine the within-participants outcomes of night shifts vs regular day shifts, with the individual participants serving as their own baseline. Second, participants who joined the study were possibly more motivated or more able to cope with the work pressures than those who did not sign up or those who adhere to the study procedures. The flexible timing and unsupervised nature of the EMA assessments may also have introduced additional variance. Third, light exposure, whose timing and intensity could influence circadian timing and outcomes of interest, was not measured. While it might not be possible to control all influential factors in field studies, the outcomes of timing were well accounted for in control analyses.

## Conclusions

In this cohort study, a float schedule, where physicians worked 12-hour night shifts concentrated into 5 to 7 consecutive shifts, seemed to offer benefits over a traditional call schedule for physician sleep regularity, mood, and cognitive performance. Vigilance after night shifts, irrespective of schedule, was higher when naps were taken.

## Notes

Supplement 1. **eFigure 1. **Construction of the Integrated Sleep Timeline From Time-Use Diary Inputs, and Device Activity Data (MET) and Device Sleep Data (Hypnogram) **eFigure 2. **Cognitive Assessments as Implemented in the Daily Ecological Momentary Assessment (EMA) Smartphone Application **eFigure 3. **EMA Session Completion Timings for Regular Days (Top Panel) and Night Shift Days (Bottom Panel) **eTable 1. **Linear Mixed Model Analysis of Daily EMA Sleep Ratings **eTable 2. **Pairwise Comparisons **eTable 3. **Linear Mixed Model Analysis of Daily EMA Mood and Motivation Ratings **eTable 4. **Pairwise Comparisons **eTable 5. **Linear Mixed Model Analysis of Daily EMA Stress and Loneliness Ratings **eTable 6. **Pairwise Comparisons **eTable 7. **Linear Mixed Model Analysis of Daily EMA Cognitive Assessment **eTable 8. **Pairwise Comparisons **eTable 9. **Preshift and Postshift Ratings of Mood and Motivation **eTable 10. **Preshift and Postshift Ratings of Sleep Quality and Sleepiness **eFigure 4. **Preshift and Postshift Sleep and Wellbeing Ratings **eTable 11. **Preshift and Postshift Performance on the 3-Minute PVT-B **eFigure 5. **Preshift and Postshift Vigilance Performance **eTable 12.** Performance on the 3-Minute PVT-B After Night Shifts With and Without a Nap **eFigure 6. **Postshift Vigilance Performance After Night Shifts With and Without a Nap **eTable 13. **Linear Mixed Models for 10-Minute PVT Performance **eFigure 7. **10-Minute PVT Performance on Control Days and Postnight Shift Days for the Float and Call Groups

Supplement 2.
Data Sharing Statement
