# Supplementary material for: Sleep, Well-Being, and Cognition in Medical Interns on a Float or Overnight Call Schedule
Source: JAMA Netw Open. 2024 Oct 11;7(10):e2438350. doi: 10.1001/jamanetworkopen.2024.38350 (PMC11581674; doi:10.1001/jamanetworkopen.2024.38350)
Supplement: Supplement 1. — eFigure 1. Construction of the Integrated Sleep Timeline From Time-Use Diary Inputs, and Device Activity Data (MET) and Device Sleep Data (Hypnogram) eFigure 2. Cognitive Assessments as Implemented in the Daily Ecological Momentary Assessment (EMA) Smartphone Application eFigure 3. EMA Session Completion Timings for Regular Days (Top Panel) and Night Shift Days (Bottom Panel) eTable 1. Linear Mixed Model Analysis of Daily EMA Sleep Ratings eTable 2. Pairwise Comparisons eTable 3. Linear Mixed Model Analysis of Daily EMA Mood and Motivation Ratings eTable 4. Pairwise Comparisons eTable 5. Linear Mixed Model Analysis of Daily EMA Stress and Loneliness Ratings eTable 6. Pairwise Comparisons eTable 7. Linear Mixed Model Analysis of Daily EMA Cognitive Assessment eTable 8. Pairwise Comparisons eTable 9. Preshift and Postshift Ratings of Mood and Motivation eTable 10. Preshift and Postshift Ratings of Sleep Quality and Sleepiness eFigure 4. Preshift and Postshift Sleep and Wellbeing Ratings eTable 11. Preshift and Postshift Performance on the 3-Minute PVT-B eFigure 5. Preshift and Postshift Vigilance Performance eTable 12. Performance on the 3-Minute PVT-B After Night Shifts With and Without a Nap eFigure 6. Postshift Vigilance Performance After Night Shifts With and Without a Nap eTable 13. Linear Mixed Models for 10-Minute PVT Performance eFigure 7. 10-Minute PVT Performance on Control Days and Postnight Shift Days for the Float and Call Groups [file jamanetwopen-e2438350-s001.pdf]

Supplemental Online Content

Massar SAA, Chua XY, Leong R, et al. Sleep, well-being, and cognition in medical interns on a float or overnight-call schedule. *JAMA Netw Open*. 2024;7(10):e2438350. doi:10.1001/jamanetworkopen.2024.38350

eFigure 1. Construction of the Integrated Sleep Timeline From Time-Use Diary Inputs, and Device Activity Data (MET) and Device Sleep Data (Hypnogram)
eFigure 2. Cognitive Assessments as Implemented in the Daily Ecological Momentary Assessment (EMA) Smartphone Application
eFigure 3. EMA Session Completion Timings for Regular Days (Top Panel) and Night Shift Days (Bottom Panel)
eTable 1. Linear Mixed Model Analysis of Daily EMA Sleep Ratings
eTable 2. Pairwise Comparisons
eTable 3. Linear Mixed Model Analysis of Daily EMA Mood and Motivation Ratings
eTable 4. Pairwise Comparisons
eTable 5. Linear Mixed Model Analysis of Daily EMA Stress and Loneliness Ratings
eTable 6. Pairwise Comparisons
eTable 7. Linear Mixed Model Analysis of Daily EMA Cognitive Assessment
eTable 8. Pairwise Comparisons
eTable 9. Preshift and Postshift Ratings of Mood and Motivation
eTable 10. Preshift and Postshift Ratings of Sleep Quality and Sleepiness
eFigure 4. Preshift and Postshift Sleep and Wellbeing Ratings
eTable 11. Preshift and Postshift Performance on the 3-Minute PVT-B
eFigure 5. Preshift and Postshift Vigilance Performance
eTable 12. Performance on the 3-Minute PVT-B After Night Shifts With and Without a Nap
eFigure 6. Postshift Vigilance Performance After Night Shifts With and Without a Nap
eTable 13. Linear Mixed Models for 10-Minute PVT Performance
eFigure 7. 10-Minute PVT Performance on Control Days and Postnight Shift Days for the Float and Call Groups

This supplemental material has been provided by the authors to give readers additional information about their work.

© 2024 Massar SAA et al. *JAMA Network Open*.

Integrated sleep timeline

As data from consumer wearables are not always optimized for detecting irregular sleep or short episodes of sleep, recordings from the Oura ring were triangulated with daily time-use diary inputs. Participants were requested to fill their daily activities in an electronic diary implemented in the smartphone research app. While they were able to fill in multiple activity categories, they were encouraged to pay specific attention to their sleep, work, and meal timings. By combining data from the Oura-derived sleep recordings, Oura-derived activity recordings, and the electronic time-use diary inputs, we were able to i) impute missing data from either channel, and ii) arbitrate when there was discrepancy between different channel outputs (e.g., using time-use diary to confirm sleep or wake, when the Oura ring recorded low activity but did not register sleep). This allowed us to create an integrated timeline of moment-to-moment probability that the person was awake or asleep given the different data channels.

For each of the three data streams, a timeseries of sleep probability was first generated for each subject. Firstly, Oura-derived sleep hypnograms from all recorded sleep periods, both main sleep and recorded naps, were collated and converted into a 5-minute sleep probability timeseries ranging from 1 (sleep state) to 0 (wake state). Secondly, 1-minute metabolic-equivalent (MET) readings, a measure of activity level obtained from Oura, was recoded such that the minimum activity levels (MET = 0.9) indicated likely sleep state = 1, low activity levels (MET = 1.0) as uncertain (0.5), and active periods (MET ≥ 1.1) as likely wake (0). The output was then regrouped into 5-minute bins by taking the average of the new window to form an activity-based sleep probability timeseries. Periods of non-wear were treated as missing for both Oura-derived data streams. Lastly, for the time-use diary, a 5-minute sleep probability timeseries was generated by assigning a value of 1 for intervals self-reported as "Sleep", and 0 for all other reported activities. Intervals without a recorded activity were treated as missing.

eFigure 1. Construction of the Integrated Sleep Timeline From Time-Use Diary Inputs, and Device Activity Data (MET) and Device Sleep Data (Hypnogram)

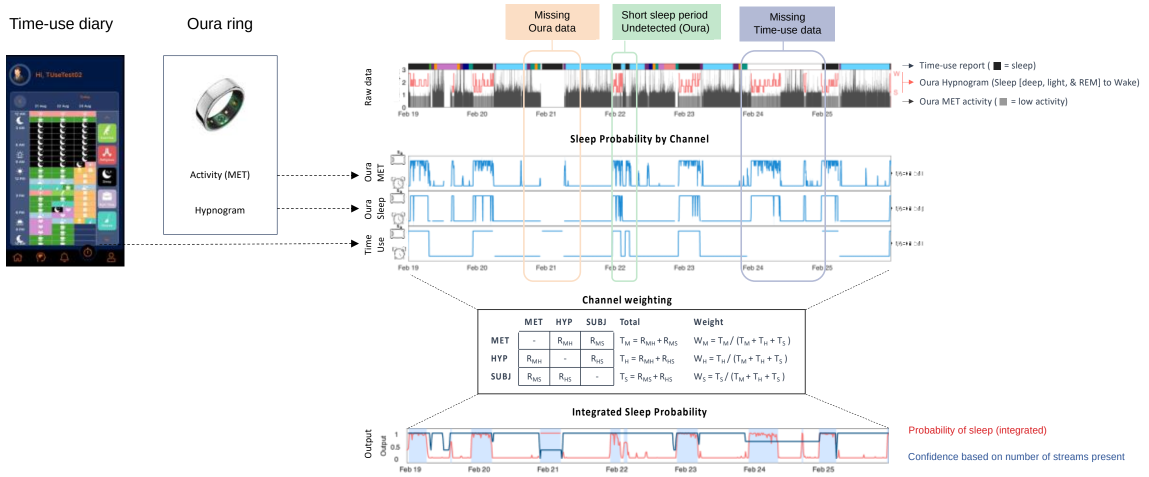

© 2024 Massar SAA et al. *JAMA Network Open*.

For each of the three data streams, a timeseries of sleep probability ($P_{i,t}$) was first generated for each subject. Firstly, Oura-derived sleep hypnograms from all recorded sleep periods, both main sleep and recorded naps, were collated and converted into a 5-minute sleep probability timeseries ranging from $P_{i,t} = 1$ (sleep state) to $P_{i,t} = 0$ (wake state). Secondly, 1-minute metabolic-equivalent (MET) readings, a measure of activity level obtained from Oura, was recoded such that the minimum activity levels (MET = 0.9) indicated likely sleep state $P_{i,t} = 1$, low activity levels (MET = 1.0) as uncertain ($P_{i,t} = 0.5$), and active periods (MET ≥ 1.1) as likely wake ($P_{i,t} = 0$). The output was then regrouped into 5-minute bins by taking the average of the new window to form an activity-based sleep probability timeseries. Periods of non-wear were treated as missing for both Oura-derived data streams. Lastly, for the time-use diary, a 5-minute sleep probability timeseries was generated by assigning a value of $P_{i,t} = 1$ for intervals self-reported as "Sleep", and $P_{i,t} = 0$ for all other reported activities. Intervals without a recorded activity were treated as missing.

For each stream, a correlation index was then calculated by summing the correlation coefficients obtained through correlating each timeseries with the remaining two.

$$Summed\ stream\ correlation: T_i = r_{i*j} + r_{i*k}$$

The final weight for each stream was determined by dividing its index with the sum of all three indices resulting in a total weight of 1.

$$Stream\ weight: W_i = \frac{T_i}{(T_i + T_j + T_k)}$$

Each sleep probability timeseries was then multiplied by their corresponding weight and summed to form an integrated timeseries. The integrated timeseries was subsequently reweighted based on the available streams at each epoch – dividing the summed value by the total weight contributing to each epoch – resulting in a final 5-minute sleep probability timeseries with values ranging from 0 to 1. Epochs without any contributing data streams were labelled as missing.

$$Integrated\ sleep\ probability: P_t = \frac{\sum_{i \in M_t} W_i P_{i,t}}{\sum_{i \in M_t} W_i}$$

Where:
M_t is the set of available streams

For sleep/wake state determination, the 5-minute sleep probability was then regrouped into 15-minute intervals by taking the average of the new window and then binarized to a final label of 1 indicating sleep state (probability > 0.5), and 0 indicating wake state (probability ≤ 0.5). For each day, two 12h sleep duration (nocturnal: previous day 8PM – current day 8AM; daytime: current day 8AM – 8PM) were then calculated. For each of these 12h periods, only days with less than 25% missing data from the final integrated timeseries were included for further analyses.

© 2024 Massar SAA et al. *JAMA Network Open*.

Ecological Momentary Assessment (EMA)

Cognitive assessment

In the same EMA session, participants completed three cognitive tasks (see eFigure 2). The Symbol Search task, assessing speed of processing; the Dot Memory task, assessing spatial working memory[22]; and the 3-minute psychomotor vigilance task (PVT), assessing vigilant attention[23].

For the Symbol Search task, participants were shown three pairs of symbols on the top half of the screen and two pairs at the bottom. Participants tapped on the pair below that matched one of the pairs on top as fast as possible. Each session consisted of 12 trials. The median response time of correct trials was used to assess perceptual speed. Sessions with fewer than 10 responded trials, or fewer than 6 correct trials were considered to not be performed according to instructions and excluded from analyses.

In the Dot Memory task (adapted from[22]) participants were shown a 5-by-5 grid with 3 randomly placed orange dots. They had to remember the position of the dots. After 3 seconds, a distraction screen was presented displaying letters (“E”s and “F”s) and participants were instructed to tap on all the “F”s. Finally, an empty 5-by-5 grid was presented, and participants had to tap on the position of the three dots shown on the first screen. Each session consisted of four trials, and scores were calculated based on the distance between the positions of the presented dots and the responses given. The sum across all trials (maximum 48) was used as a measure of spatial working memory. Trials with fewer than half of the available Fs tapped, or more than two Es tapped were considered invalid. Only sessions with all four valid trials were included in the analysis.

The Psychomotor Vigilance Test (PVT) is considered the gold standard behavioral test for alertness. In the EMA app an abbreviated version was implemented (3-minute PVT-B). Each trial started with the presentation of a dot and a button. At random intervals (1-4s), the dot was replaced by a running timer and participants had to respond as quickly as possible by button press. Upon button press, reaction time was shown on screen and the next trial started. If no response was detected after 9999ms, the trial ended as a non-response. Responses detected prior to the start of the timer, or with a reaction time under 150ms, were considered false starts. As a measure of vigilance, median reaction time was calculated based on valid trials after excluding non-response and false start trials. Trials with RT greater than 500ms were considered lapses. Session consisted of around 45 trials depending on participant performance. Session with fewer than 25 total trials or with a high number of false start or lapse trials (≥50% of total trials) were considered non-valid and were excluded from analyses. Participants with average performance scores across sessions below 75% were withdrawn from analyses due to their consistent poor performance and possible noncompliance with task instructions.

eFigure 2. Cognitive Assessments as Implemented in the Daily Ecological Momentary Assessment (EMA) Smartphone Application

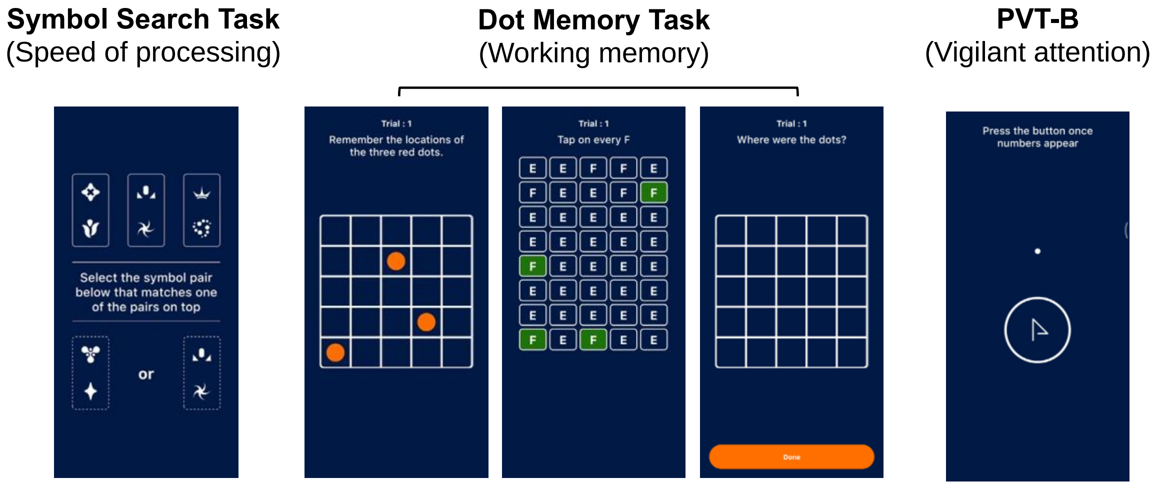

© 2024 Massar SAA et al. *JAMA Network Open*.

EMA timing
To allow for completion on irregular work schedules, participants were able to complete the EMA session once a day between 5am and 10pm. They were instructed to complete the session at the time most suited to their schedule, but preferably about 30 minutes after waking up. Consequently, most EMA's were completed in the morning before work on regular days, and in the daytime after the work shift on night-shift days (reflecting post-night shift wellbeing/performance). Most participants completed the sessions between 5 and 7am on regular days (before their work shift). On night shift days, EMA sessions were most often completed in the morning after the night shift had ended, or in the afternoon before the start of their night shift (see eFigure 3).

eFigure 3. EMA Session Completion Timings for Regular Days (Top Panel) and Night Shift Days (Bottom Panel).

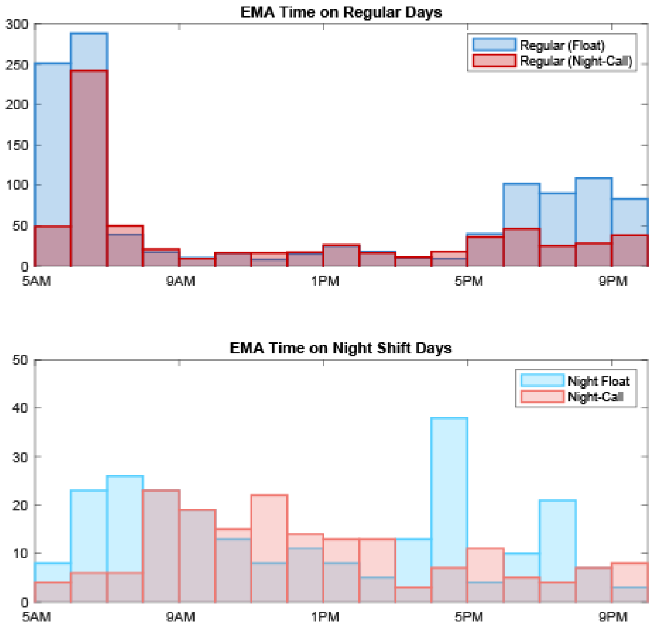

Control Analyses

Control analysis 1: Controlling for prior sleep history
To control for the possible confounding effects of these timing differences, two sets of control analyses were performed. First, we ran a set of linear mixed models with two added sleep history variables included as control covariates (i.e., total sleep duration in the prior 24 hours and time awake since the last sleep episode). Results of both the main analysis (Model 1) and the control analyses including the prior sleep variables (Model 2) are displayed in eTables 1-8. Results showed that prior sleep history was significantly associated with most outcome variables. Importantly, for all variables that showed the critical Group x Shift interaction in Model 1, this interaction remained significant after controlling for prior sleep history in Model 2. Post-hoc pairwise comparisons confirmed that all effects indicated poorer outcomes (i.e. sleep quality, sleepiness, mood, motivation, Dot memory task, 3-min PVT) for the call group after their night shift (all ps' < .03), with no regular versus night shift deterioration for the float group.

© 2024 Massar SAA et al. *JAMA Network Open*.

eTable 1. Linear Mixed Model Analysis of Daily EMA Sleep Ratings

	Model 1[a]			Model 2[b]		
**Sleep Quality**	F	p	Estimate	F	p	Estimate
Intercept	5.11	0.03	3.24	3.22	0.08	2.56
Group	17.07	<.001	-0.01	11.16	0.00	-0.04
Shift	175.97	<.001	-0.09	55.83	<.001	-0.02
**Group x Shift***	**120.32**	**<.001**	**-0.89**	**56.14**	**<.001**	**-0.65**
Sex	0.42	0.52	-0.08	0.22	0.64	-0.06
Age	0.00	0.99	0.00	0.00	0.97	0.00
BMI	0.23	0.63	0.01	0.25	0.62	0.01
Day-in-Study	2.16	0.14	0.00	0.69	0.41	0.00
Sleep history (prior 24h)	-	-	-	73.10	<.001	0.00
Time since wake	-	-	-	0.96	0.33	0.00
**Sleepiness**	F	p	Estimate	F	p	Estimate
Intercept	0.56	0.46	14.41	1.26	0.27	26.71
Group	13.9	<.001	1.52	6.69	0.01	2.16
Shift	74.87	<.001	1.13	10.53	0.001	-0.18
**Group x Shift***	**56.5**	**<.001**	**14.83**	**13.56**	**<.001**	**8.06**
Sex	1.34	0.25	3.19	0.97	0.33	2.66
Age	2.75	0.1	1.76	3.12	0.08	1.85
BMI	0.45	0.51	-0.25	0.47	0.5	-0.26
Day-in-Study	0.58	0.45	0.02	2.1	0.15	0.03
Sleep history (prior 24h)	-	-	-	59.31	<.001	-0.04
Time since wake	-	-	-	0.31	0.58	0

[a] Model 1 controls for demographics and day-in-study
[b] Model 2 controls for demographics, day-in-study, and prior sleep history and time since wake from last sleep episode
** Bold faced text indicates that the critical Group x Shift interaction is significant at adjusted p-value <0.05 using the Benjamini-Hochberg method*

eTable 2. Pairwise Comparisons

	Model 1			*Model 2*		
**Sleep Quality**	Regular Shift	Night Shift	p-value	Regular Shift	Night Shift	p-value
Call-Group	**3.4 ± .08**	**2.4 ± .10**	**< .001**	**3.4 ± .08**	**2.7 ± .10**	**< .001**
Float-group	3.4 ± .07	3.3 ± .08	.056	3.4 ± .07	3.4 ± .08	.67
**Sleepiness**						
Call-Group	**56.0 ± 1.8**	**72.0 ± 2.1**	**< .001**	**57.1 ± 1.8**	**65.0 ± 2.3**	**< .001**
Float-group	54.5 ± 1.5	55.6 ± 1.8	.38	54.9 ± 1.4	54.7 ± 1.8	.89

© 2024 Massar SAA et al. *JAMA Network Open*.

eTable 3. Linear Mixed Model Analysis of Daily EMA Mood and Motivation Ratings

	Model 1[a]			Model 2[b]		
**Mood**	F	p	Estimate	F	p	Estimate
Intercept	0.002	0.96	1.13	0.001	0.98	-1.87
Group	0.72	0.4	1.3	0.32	0.58	0.79
Shift	17.19	<.001	-0.23	2.23	0.14	0.62
**Group x Shift***	**15.05**	**<.001**	**-6.56**	**5.17**	**0.02**	**-4.3**
Sex	0.93	0.34	2.62	1.09	0.3	2.9
Age	1.46	0.23	1.26	1.1	0.3	1.13
BMI	5.27	0.02	0.86	4.9	0.03	0.85
Day-in-Study	28.66	<.001	-0.1	23.62	<.001	-0.1
Sleep history (prior 24h)	-	-	-	12.41	<.001	0.01
Time since wake	-	-	-	1.69	0.19	0.002

**Motivation**	F	p	Estimate	F	p	Estimate
Intercept	0.32	0.57	18.78	0.24	0.63	15.34
Group	0.58	0.45	2.25	0.05	0.83	1.75
Shift	51.72	<.001	-1.78	10.26	0.001	-0.86
**Group x Shift***	**25.45**	**<.001**	**-8.31**	**6.39**	**0.01**	**-4.62**
Sex	0.001	0.98	-0.07	0.02	0.9	0.39
Age	0.06	0.82	0.26	0.01	0.94	0.08
BMI	6.12	0.02	0.99	5.86	0.02	0.99
Day-in-Study	10.31	0.001	-0.06	9.17	0.002	-0.06
Sleep history (prior 24h)	-	-	-	28.83	<.001	0.02
Time since wake	-	-	-	1.97	0.16	-0.002

[a] *Model 1 controls for demographics and day-in-study*

[b] *Model 2 controls for demographics, day-in-study, and prior sleep history and time since wake from last sleep episode*

** Bold faced text indicates that the critical Group x Shift interaction is significant at adjusted p-value <0.05 using the Benjamini-Hochberg method*

eTable 4. Pairwise Comparisons

	Model 1			*Model 2*		
**Mood**	Regular Shift	Night Shift	p-value	Regular Shift	Night Shift	p-value
Call-Group	**50.8 ± 1.8**	**44.1 ± 2.0**	**< .001**	**50.0 ± 1.8**	**46.3 ± 2.2**	**.023**
Float-group	49.5 ± 1.4	49.3 ± 1.7	.83	49.2 ± 1.5	49.8 ± 1.7	.59
**Motivation**						
Call-Group	**47.4 ± 1.9**	**37.3 ± 2.1**	**< .001**	**46.8 ± 2.0**	**41.3 ± 2.3**	**< .001**
Float-group	45.2 ± 1.5	43.4 ± 1.8	.097	45.0 ± 1.6	44.2 ± 1.8	.43

© 2024 Massar SAA et al. *JAMA Network Open.*

eTable 5. Linear Mixed Model Analysis of Daily EMA Stress and Loneliness Ratings

	Model 1[a]			Model 2[b]		
**Stress**	F	p	Estimate	F	p	Estimate
Intercept	0.68	0.41	35.35	0.56	0.46	32.34
Group	0.01	0.92	1.26	0.004	0.95	0.93
Shift	24.85	<.001	-3.53	16.67	<.001	-3.66
Group x Shift	1.07	0.3	-1.85	0.52	0.47	-1.43
Sex	0	0.99	-0.02	0.06	0.81	0.96
Age	0.09	0.77	0.45	0.12	0.73	0.54
BMI	0.01	0.91	-0.06	0.002	0.97	-0.02
Day-in-Study	1.31	0.25	-0.02	1.3	0.25	-0.02
Sleep history (prior 24h)	-	-	-	0.003	0.96	0
Time since wake	-	-	-	0.67	0.41	-0.001
**Loneliness**	F	p	Estimate	F	p	Estimate
Intercept	2.03	0.16	68.05	2.15	0.15	71.28
Group	0.14	0.71	2.38	0.07	0.8	2.16
Shift	0.26	0.61	1.35	0.1	0.76	1.43
Group x Shift	1.25	0.26	-1.86	1.43	0.23	-2.23
Sex	0.61	0.44	-3.6	0.52	0.47	-3.39
Age	0.32	0.57	-1.02	0.38	0.54	-1.12
BMI	20	0.65	-0.29	0.17	0.69	-0.26
Day-in-Study	3.25	0.07	0.03	0.71	0.4	0.02
Sleep history (prior 24h)	-	-	-	0.26	0.61	-0.002
Time since wake	-	-	-	0.5	0.48	0

[a] *Model 1 controls for demographics and day-in-study*

[b] *Model 2 controls for demographics, day-in-study, and prior sleep history and time since wake from last sleep episode*

eTable 6. Pairwise Comparisons

	Model 1			*Model 2*		
**Stress**	Regular Shift	Night Shift	p-value	Regular Shift	Night Shift	p-value
Call-Group	45.8 ± 2.6	40.47 ± 2.8	< .001	45.7 ± 2.6	40.6 ± 2.9	.003
Float-group	44.6 ± 2.1	41.1 ± 2.3	.002	44.7 ± 2.1	41.1 ± 2.3	.002
**Loneliness**						
Call-Group	38.8 ± 3.0	37.6 ± 3.1	.69	38.3 ± 3.1	37.5 ± 3.3	.62
Float-group	35.8 ± 2.4	37.1 ± 2.6	.21	36.1 ± 2.5	37.6 ± 2.6	.20

© 2024 Massar SAA et al. *JAMA Network Open.*

eTable 7. Linear Mixed Model Analysis of Daily EMA Cognitive Assessment

	Model 1[a]			Model 2[b]		
**Symbol search**	F	p	Estimate	F	p	Estimate
Intercept	0.54	0.46	-464.11	0.61	0.44	-490.76
Group	0.68	0.41	24.53	0.33	0.57	10.38
Shift	0.91	0.34	-26.02	1.21	0.27	-34.16
Group x Shift	0.98	0.32	26.51	1.27	0.26	33.14
Sex	3.98	0.05	107.1	4.08	0.05	110.86
Age	7.14	0.01	55.66	6.96	0.01	56.21
BMI	7.06	0.1	19.67	7.55	0.01	20.77
Day-in-Study	76.28	<.001	-2.64	46.91	<.001	-2.14
Sleep history (prior 24h)				0.9	0.34	-0.06
Time since wake				0.81	0.37	-0.02
**Dot Memory**	F	p	Estimate	F	p	Estimate
Intercept	22.37	<.001	49.56	20.39	<.001	50.57
Group	2.89	0.09	-0.75	3.26	0.07	-0.84
Shift	1.99	0.16	0.31	1.12	0.29	0.46
**Group x Shift***	**6.34**	**0.01**	**-1.42**	**6.88**	**0.01**	**-1.64**
Sex	2.02	0.16	-1.42	1.48	0.23	-1.29
Age	0.34	0.56	-0.23	0.39	0.54	-0.26
BMI	0.26	0.61	-0.07	0.21	0.65	-0.07
Day-in-Study	53.82	<.001	0.05	47.98	<.001	0.05
Sleep history (prior 24h)				0.13	0.72	0
Time since wake				0.49	0.49	0
**3-min PVT**	F	p	Estimate	F	p	Estimate
Intercept	10.85	0.001	242.96	12.01	<.001	257.11
Group	3.89	0.05	2.32	1.62	0.21	1.93
Shift	41.25	<.001	-0.33	4.21	0.04	-2.4
**Group x Shift***	**44.03**	**<.001**	**21.01**	**12.81**	**<.001**	**12.66**
Sex	2.36	0.13	11.61	3.2	0.08	13.43
Age	0.004	0.95	0.19	0.02	0.9	0.38
BMI	2.16	0.15	1.53	2.06	0.16	1.48
Day-in-Study	154.84	<.001	0.44	135.52	<.001	0.43
Sleep history (prior 24h)				24.37	<.001	-0.04
Time since wake				10.13	0.001	-0.01

[a] *Model 1 controls for demographics and day-in-study*

[b] *Model 2 controls for demographics, day-in-study, and prior sleep history and time since wake from last sleep episode last sleep episode*

** Bold faced text indicates that the critical Group x Shift interaction is significant at adjusted p-value <0.05 using the Benjamini-Hochberg method*

© 2024 Massar SAA et al. *JAMA Network Open.*

eTable 8. Pairwise Comparisons

	Model 1			*Model 2*		
**Symbol Search**	Regular Shift	Night Shift	p-value	Regular Shift	Night Shift	p-value
Call-Group	1348.6 ± 34.9	1349.1 ± 38.3	.98	1329.3 ± 36.0	1328.2 ± 40.9	.97
Float-group	1324.1 ± 28.3	1298.1 ± 31.5	.14	1318.9 ± 28.9	1284.7 ± 32.1	.054
**Dot Memory**						
Call-Group	**42.2 ± .6**	**41.1 ± .7**	**.01**	**42.2 ± .70**	**41.0 ± .82**	**.028**
Float-group	43.0 ± .5	43.3 ± .6	.39	43.0 ± .56	43.5 ± .63	.22
**3-min PVT**						
Call-Group	**300.6 ± 5.0**	**321.3 ± 5.3**	**< .001**	**301.8 ± 5.0**	**312.0 ± 5.5**	**< .001**
Float-group	298.3 ± 4.0	298.0 ± 4.3	.87	299.8 ± 4.0	297.4 ± 4.3	.43

© 2024 Massar SAA et al. *JAMA Network Open*.

Control analysis 2: EMA assessments before and after work shifts

A secondary question is whether there were any differences in subjective readiness (mood, motivation, sleepiness), and cognitive performance (dot memory, 3-min PVT), at the start of each of the different work shifts (i.e. does the schedule type affect how rested physicians are when they start their working day), or whether the observed differences developed over the course of the working day. In order to test these questions, EMA sessions were selected based on whether they were completed prior to the shift start time (pre-shift), or at the end of the shift (post-shift). Separate Linear Mixed Models were run for the pre-shift sessions and post-shift sessions with Group (Call, Float) and Shift (Regular shift, Night shift) as factors. Demographics (Age, Gender, BMI) and day-in-study were entered as control variables.

For mood and motivation ratings, the Group x Shift interactions were significant in the post-shift time window but not in the pre-shift window (eTable 9). Post-hoc pairwise comparisons (see eFigure 4) indicated that the call group participants were in poorer mood ($p = .004$) and had lower motivation ($p < .001$) after their night shift, but not the float group (p's > .47). Similarly, a Group x Shift interaction was found for sleep quality in the post-shift but not the pre-shift window (eTable 10). Pairwise comparisons showed that both groups rated poorer sleep quality after their night shift compared to their regular shifts (eFigure 4), but this difference was smaller for the float group (post-night shift: 3.2 ± .1, post-regular shift: 3.4 ± .09, $p = .026$) than for the call group (post-night shift: 2.6 ± .1, post-regular shift: 3.5 ± .1, $p < .001$). For sleepiness, a Group x Shift interaction was found in both the pre-shift and post-shift windows (eTable 10). Pre-shift, the float group had lower sleepiness scores during their float week (47.6 ± 2.5) compared to their regular shifts (55.0 ± 1.8, $p = .001$, see eFigure 4). Whereas there were no pre-shift differences for the call group ($p = .58$). On the other hand, post-shift both groups showed an increase in sleepiness after night shifts compared to regular shifts. This difference was smaller for the float group cc

© 2024 Massar SAA et al. *JAMA Network Open.*

eTable 9. Preshift and Postshift Ratings of Mood and Motivation

	Pre-shift			*Post-shift*		
**Mood**	F	p	Estimate	F	p	Estimate
Intercept	0.78	0.38	30.31	0.56	0.46	26.21
Group	0.01	0.92	-2.37	0.29	0.59	-2.67
Shift	1.15	0.29	-3.44	4.91	0.03	-7.72
**Group x Shift**	2.77	0.10	4.17	**6.41**	**0.01***	**8.22**
Sex	0.01	0.94	0.24	0.25	0.62	1.54
Age	0.00	0.96	-0.07	0.54	0.47	0.89
BMI	6.99	0.01	1.17	0.23	0.64	0.20
Day-in-Study	17.61	<.001	-0.12	3.35	0.07	-0.06
**Motivation**	F	p	Estimate	F	p	Estimate
Intercept	0.69	0.41	28.81	0.34	0.56	29.65
Group	0.01	0.92	-1.36	0.12	0.73	-9.21
Shift	0.10	0.76	-1.44	19.20	<.001	-15.17
**Group x Shift**	0.81	0.37	2.14	**27.07**	**<.001***	**16.46**
Sex	0.04	0.84	0.70	0.91	0.34	-3.15
Age	0.06	0.81	-0.31	0.22	0.64	0.61
BMI	7.35	0.01	1.25	0.83	0.37	0.42
Day-in-Study	4.33	0.04	-0.05	3.73	0.05	-0.06

** Bold faced text indicates that the critical Group x Shift interaction is significant at adjusted p-value <0.05 using the Benjamini-Hochberg method*

eTable 10. Preshift and Postshift Ratings of Sleep Quality and Sleepiness

	Pre-shift			*Post-shift*		
**Sleep Quality**	F	p	Estimate	F	p	Estimate
Intercept	5.72	0.02	3.61	0.77	0.38	1.78
Group	0.02	0.90	-0.04	4.74	0.03	-0.06
Shift	0.10	0.75	-0.08	53.26	<.001	-0.89
**Group x Shift**	1.11	0.29	0.12	**24.16**	**<.001***	**0.72**
Sex	0.35	0.56	-0.09	0.17	0.68	-0.07
Age	0.00	0.98	0.00	0.52	0.47	0.04
BMI	0.31	0.58	-0.01	1.82	0.18	0.03
Day-in-Study	6.78	0.01	0.00	0.28	0.60	0.00
**Sleepiness**	F	p	Estimate	F	p	Estimate
Intercept	0.41	0.53	21.29	0.12	0.74	4.65
Group	4.15	0.04	-1.59	7.73	0.01	-1.66
Shift	5.13	0.02	0.95	45.60	<.001	17.78
**Group x Shift**	**8.77**	**0.00***	**-8.39**	**11.32**	**<.001***	**-11.81**
Sex	0.71	0.40	2.75	0.72	0.40	2.64
Age	1.98	0.16	1.70	2.08	0.15	1.78
BMI	0.71	0.40	-0.36	0.49	0.49	0.30
Day-in-Study	0.00	0.95	0.00	0.04	0.85	0.01

** Bold faced text indicates that the critical Group x Shift interaction is significant at adjusted p-value <0.05 using the Benjamini-Hochberg method*

© 2024 Massar SAA et al. *JAMA Network Open*.

eFigure 4. Preshift and Postshift Sleep and Wellbeing Ratings

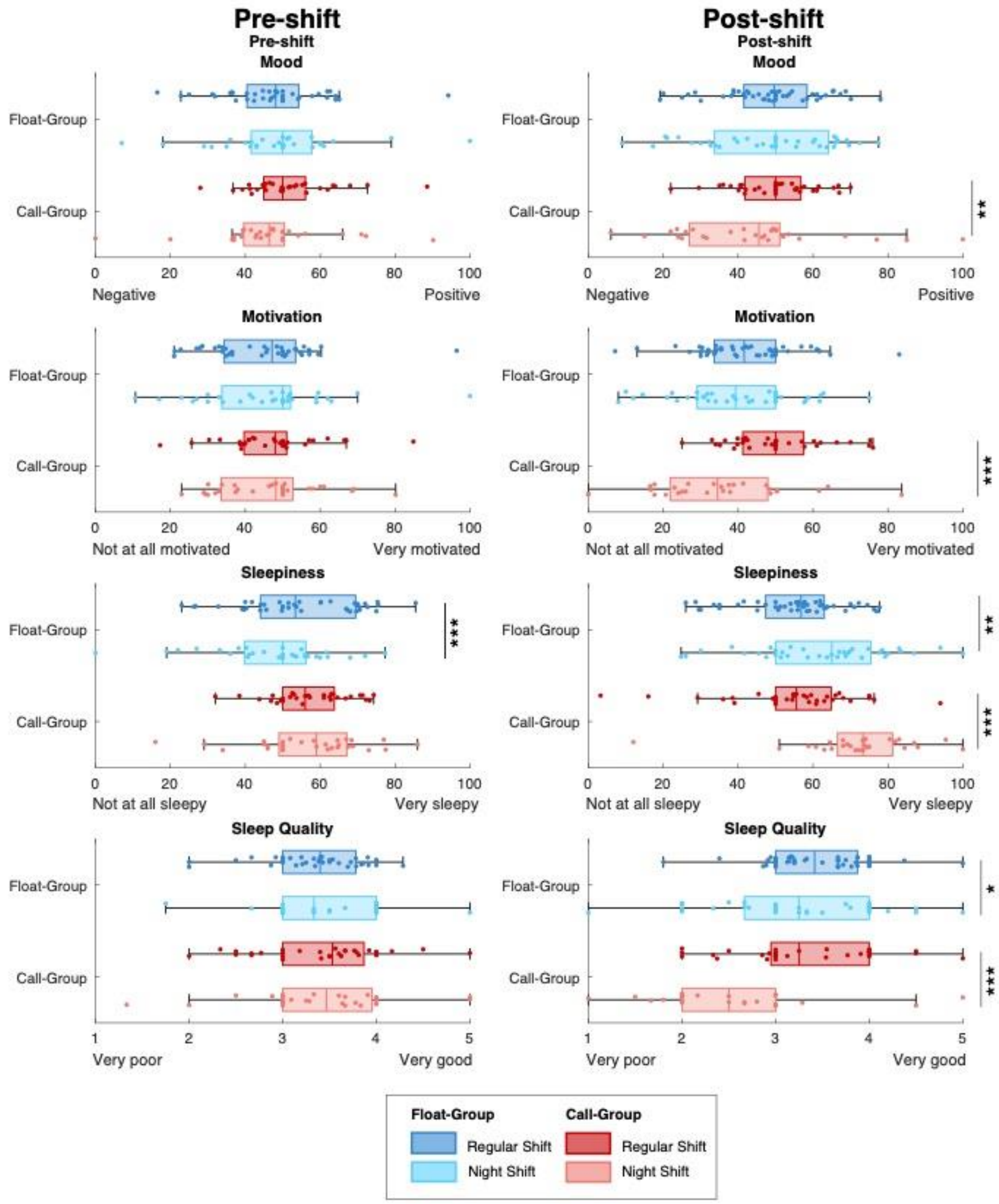

© 2024 Massar SAA et al. *JAMA Network Open*.

eTable 11. Preshift and Postshift Performance on the 3-Minute PVT-B

	Pre-shift			*Post-shift*		
**Dot Memory**	F	p	Estimate	F	p	Estimate
Intercept	12.78	<.001	41.54	19.93	<.001	51.29
Group	1.08	0.30	0.58	0.80	0.37	0.55
Shift	0.01	0.94	-0.44	0.11	0.75	-0.13
Group x Shift	1.14	0.29	0.95	0.34	0.56	0.60
Sex	0.55	0.46	-0.88	1.77	0.19	-1.46
Age	0.04	0.84	0.09	0.47	0.50	-0.29
BMI	0.56	0.46	-0.12	0.45	0.50	-0.10
Day-in-Study	39.73	<.001	0.06	17.24	<.001	0.04
**3-min PVT**	F	p	Estimate	F	p	Estimate
Intercept	8.83	0.00	258.45	8.43	0.01	245.63
Group	1.61	0.21	2.32	2.60	0.11	-6.76
Shift	22.66	<.001	0.65	35.74	<.001	22.78
**Group x Shift**	**25.75**	**<.001***	**-23.54**	3.00	0.08	-10.23
Sex	0.02	0.89	1.22	1.46	0.23	10.27
Age	0.03	0.88	0.51	0.00	0.97	0.14
BMI	0.69	0.41	0.96	1.71	0.19	1.54
Day-in-Study	89.30	<.001	0.48	56.98	<.001	0.46

** Bold faced text indicates that the critical Group x Shift interaction is significant at adjusted p-value <0.05 using the Benjamini-Hochberg method*

eFigure 5. Preshift and Postshift Vigilance Performance

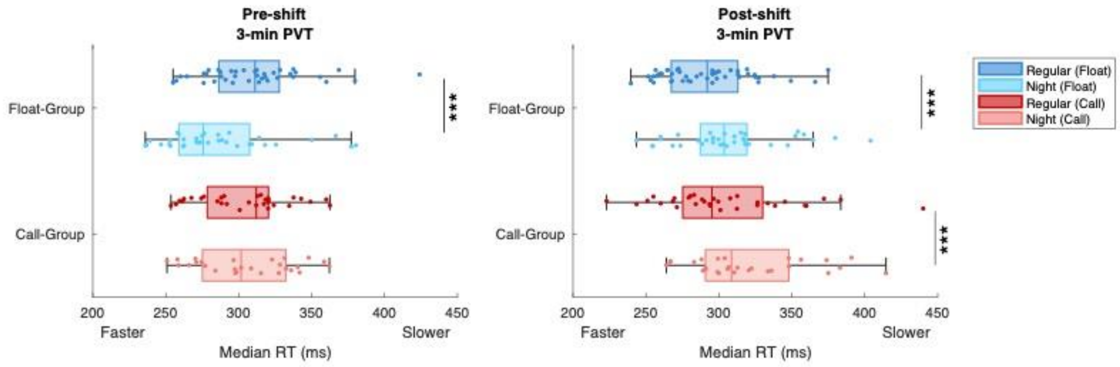

For cognitive performance, no pre-shift or post-shift differences in working memory performance were observed (eTable 11). However, for 3-min PVT performance, a pre-shift Group x Shift interaction was found, indicating that the float group performed better prior to their night-float shifts (eFigure 5; median RT: 285.0 ± 5.3) compared to their regular shifts (median RT: 307.9 ± 4.6, p < .001). No such pre-shift differences were found for the call group. In the post-shift window, both groups showed impaired PVT performance after their night shifts. Although the Group x Shift interaction did not reach significance (p = .08), this effect was numerically smaller for the float group (post-night shift: 305.6 ± 5.1, post-regular shift: 293.0 ± 4.6, p < .001) than for the call group (post-night shift: 322.5 ± 6.4, post-regular shift: 299.8 ± 6.1, p < .001).

Control analysis 3: The effect of napping on the night shift on vigilance

To examine whether naps taken on the night shift were associated with better vigilance, the 3-min PVT performance was assessed after night shifts that did include a nap and night shifts that did not include a nap (eTable 12). This analysis yielded a significant main effect of nap, but no Nap x Group interaction, suggesting that vigilance was better after a nap in both groups (eFigure 6).

eTable 12. Performance on the 3-Minute PVT-B After Night Shifts With and Without a Nap

	Post-shift		
**3-min PVT**	F	p	Estimate
Intercept	9.25	0.003	332.04
Group	4.24	0.04	14.23
**Nap**	**6.10**	**0.01**	**9.64**
Group x Nap	0.92	0.34	12.16
Sex	1.13	0.29	12.12
Age	0.30	0.59	-2.35
BMI	0.09	0.77	0.42
Day-in-Study	10.18	0.002	0.51

eFigure 6. Postshift Vigilance Performance After Night Shifts With and Without a Nap

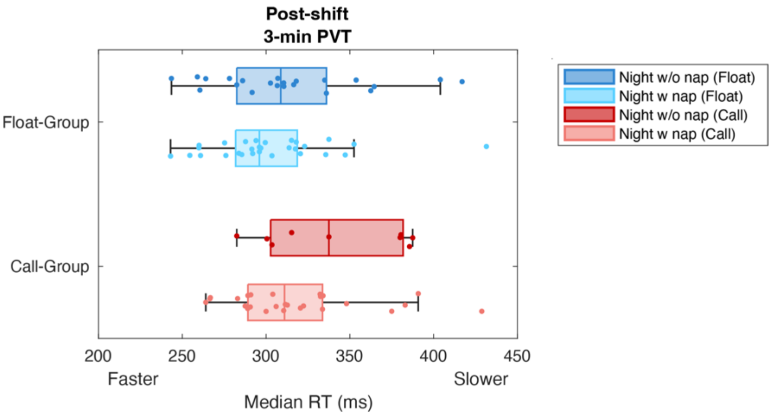

© 2024 Massar SAA et al. *JAMA Network Open*.

10-minute PVT

Given the ecological nature of the EMA assessments inter and intra-individual variance in performance might stem from extraneous factors that are not directly tied to momentary ability to perform (e.g. external noise/distraction levels, lighting conditions, body position, phone model). In order to corroborate findings from the EMA with a more standardized measurement, participants completed and additional set of standard psychomotor vigilance tests (10-minute PVT) [26].

The 10-minute PVT was performed on a laptop computer that was loaned to the participants, and participants were instructed to perform the test in a standard manner (seated upright, responding with dominant hand, in a quiet and comfortable space). Furthermore, session timing was more standardised (to be completed between 8am-2pm, after a full night of sleep [control days] or directly after night shift/before recovery sleep [night-shift days]). Participants were required to complete 3 PVT sessions on days after their night shift, and 3 control session after a full night of sleep. Test configuration was similar to the phone-based 3-minute PVT with two main exceptions (i.e. task duration was 10 minute per session and random target onset interval was 2-10 seconds).

Three outcome metrics were extracted, median RT, lapses of attention (responses > 500ms), and false starts (responses before target onset or < 150 after target onset). Sessions with false alarms more than 3 SD compared to the group mean (FA > 14) were excluded from the final analyses resulting in overall 390 sessions for the final analyses. Outcome measures were analysed using linear mixed models with Group (Call, Float) and Shift (Control, Night) as factors, while controlling for demographics (Age, Gender, BMI) and overall changes over the study period (Day-in study) see eTable 13 (Model 1).

All outcome measures showed a significant Group x Shift interaction (eTable 13). Follow-up pairwise comparisons showed that both groups had increased lapses and longer reaction time after their night shifts compared to control days (see eFigure 7). This effect was smaller for the float group (lapses: night shift-control difference = 3.97 ± 1.03, $p < .001$; median RT: night shift-control difference = 35.31 ± 12.3, $p = .005$) than for the call group (lapses: night shift-control difference = 9.53 ± 1.07, $p < .001$; median RT: night shift-control difference = 76.22 ± 12.9, $p < .001$). For false starts, only the call group showed a significant impairment after their night shift (night shift-control difference = 1.13 ± .34, $p < .001$), with no difference for the float group ($p = .32$). These effects remained largely preserved when prior sleep history variables (i.e., total sleep duration in the prior 24 hours and time awake since the last sleep episode) were included into the model as control variables (see eTable 13 [Model 2]).

© 2024 Massar SAA et al. *JAMA Network Open*.

eTable 13. Linear Mixed Models for 10-Minute PVT Performance

	Model 1[a]			Model 2[b]		
**Lapses**	F	p	Estimate	F	p	Estimate
Intercept	.15	.70	11.31	.001	.98	1.16
Group	.034	.85	3.10	.054	.82	2.19
Shift	89.80	< .001	-3.97	19.71	< .001	-3.19
**Group x Shift***	**12.87**	**< .001**	**-5.56**	**7.54**	**.006**	**-5.13**
Sex	2.18	.15	3.20	2.62	.11	3.04
Age	.24	.63	-.42	.00	.997	.003
BMI	.20	.65	.13	.59	.44	.19
Day-in-Study	17.47	< .001	.11	11.85	< .001	.097
Sleep history (prior 24h)	-	-	-	.86	.37	-.004
Time since wake	-	-	-	.25	.62	-.001
**Median RT**	F	p	Estimate	F	p	Estimate
Intercept	1.20	.28	254.59	.63	.43	169.31
Group	.56	.46	32.33	.17	.69	28.25
Shift	42.27	< .001	-35.31	9.43	.002	-25.91
**Group x Shift***	**4.89**	**.028**	**-40.92**	3.79	.053	-43.95
Sex	3.26	.076	34.94	3.62	.061	33.32
Age	.022	.88	-1.14	.11	.74	-2.37
BMI	.65	.42	2.02	1.08	.30	2.33
Day-in-Study	5.87	.016	.71	3.20	.075	.59
Sleep history (prior 24h)	-	-	-	.32	.57	-.027
Time since wake	-	-	-	.065	.78	.005
**False starts**	F	p	Estimate	F	p	Estimate
Intercept	.14	.71	1.99	.14	.71	1.68
Group	.045	.83	.56	.66	.42	.41
Shift	4.62	.032	.16	.28	.60	.95
**Group x Shift***	**7.05**	**.008**	**-1.29**	**6.84**	**.009**	**-1.48**
Sex	.10	.75	.15	.02	.90	.056
Age	.08	.78	-.055	.006	.94	-.014
BMI	1.11	.30	.065	.79	.38	.052
Day-in-Study	2.20	.14	.011	1.21	.27	.009
Sleep history (prior 24h)	-	-	-	2.56	.11	-.002
Time since wake	-	-	-	1.50	.22	.001

[a] Model 1 controls for demographics and day-in-study

[b] Model 2 controls for demographics, day-in-study, and prior sleep history and time since wake from last sleep episode

** Bold faced text indicates that the critical Group x Shift interaction is significant at adjusted p-value <0.05 using the Benjamini-Hochberg method*

© 2024 Massar SAA et al. *JAMA Network Open*.

eFigure 7. 10-Minute PVT Performance on Control Days and Postnight Shift Days for the Float and Call Groups.

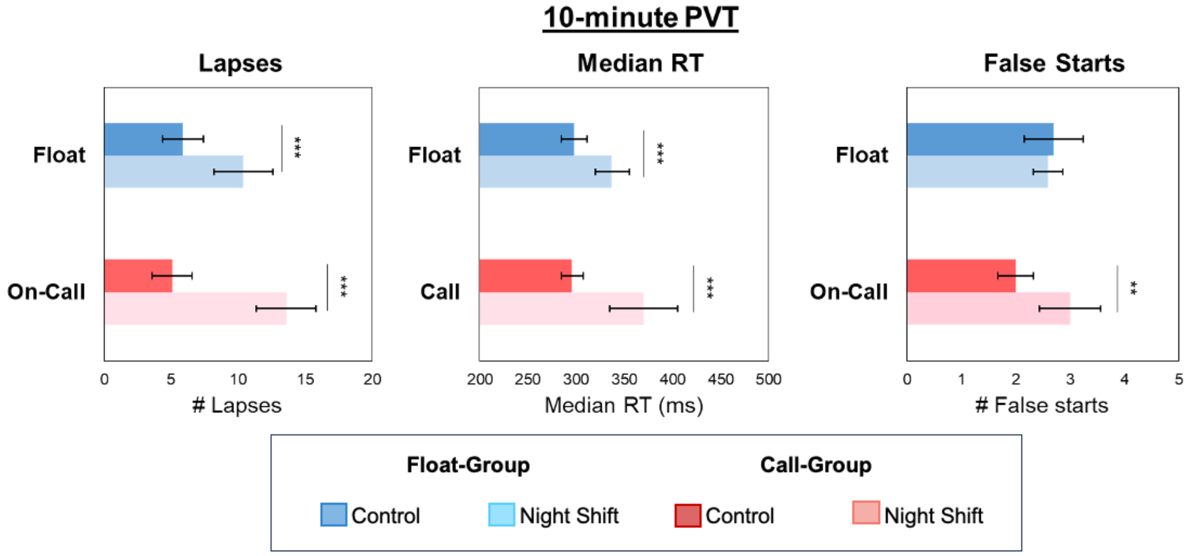

*** p < .01, *** p < .001*

© 2024 Massar SAA et al. *JAMA Network Open*.
