# Supplementary material for: Sleep, Well-Being, and Cognition in Medical Interns on a Float or Overnight Call Schedule
Source: JAMA Netw Open. 2024 Oct 11;7(10):e2438350. doi: 10.1001/jamanetworkopen.2024.38350 (PMC11581674; doi:10.1001/jamanetworkopen.2024.38350)
Supplement: Supplement 2. — Data Sharing Statement [file jamanetwopen-e2438350-s002.pdf]

Data Sharing Statement

Massar. Sleep, Well-Being, and Cognition in Medical Interns on a Float or Overnight Call Schedule. *JAMA Netw Open*. Published October 11, 2024.
doi:10.1001/jamanetworkopen.2024.38350

Data
Data available: No
